# Supplementary material for: Psychological Distress in South African Healthcare Workers Early in the COVID-19 Pandemic: An Analysis of Associations and Mitigating Factors
Source: Int J Environ Res Public Health. 2022 Aug 7;19(15):9722. doi: 10.3390/ijerph19159722 (PMC9368661; doi:10.3390/ijerph19159722)
Supplement: Supplementary file 1 [file ijerph-19-09722-s001.zip › ijerph-1829372-supplementary.pdf]

Table S1. Association between psychological distress and training, support, or appreciation.

Items	Psychological Distress Yes	Psychological Distress No	P	Effect Size g
**Training[a]**	**Mean (SD)**	**Mean (SD)**		
**7. Emergency procedures (evacuation)**	**1.58 (0.74)**	**2.08 (0.80)**	**<0.001***	**0.650**
**5. COVID-19 infection control**	**1.84 (0.61)**	**2.16 (0.51)**	**0.003***	**0.561**
6. Ventilation requirements	1.58 (0.77)	1.82 (0.79)	0.104	
4. General infection control	1.87 (0.54)	2.00 (0.49)	0.166	
3. Doffing of PPE	1.90 (0.60)	1.98 (0.52)	0.446	
2. Donning of PPE	1.93 (0.58)	1.98 (0.47)	0.595	
1. Hand hygiene	1.99 (0.40)	2.02 (0.37)	0.637	
Average score of 7 items[c]	1.18 (0.41)	2.00 (0.35)	0.009*	0.488
**Social Support[b]**				
**1. How much can each of these people be relied upon to help and support you when things get tough at work?**				
**1.1. Your immediate supervisor/boss**	**2.78 (1.06)**	**3.27 (0.75)**	**0.003***	**-0.521**
1.2. Your colleague(s)	3.07 (0.88)	3.14 (0.83)	0.683	
1.3. Your family	3.36 (0.82)	3.73 (0.70)	0.495	
1.4. Your friend(s)	3.11 (0.88)	3.29 (0.78)	0.238	
1.5. Your husband/partner	3.52 (0.94)	3.40 (1.07)	0.503	
Average score of 5 items[c]	3.23 (0.67)	3.37 (0.52)	0.215	
**Appreciation**				
**2. How often do each of these people let you know that they appreciate the job that you are doing?**				
2.1. Your immediate supervisor/boss	2.77 (1.01)	3.00 (0.94)	0.236	
2.2. Your colleague(s)	2.77 (1.06)	2.94 (0.8)	0.321	
2.3. Your family	3.49 (0.86)	3.47 (0.81)	0.924	
2.4. Your friend(s)	3.10 (0.93)	3.16 (0.93)	0.743	
2.5. Your husband/partner	3.48 (0.95)	3.44 (0.92)	0.841	
Average score of 5 items[c]	3.11 (0.74)	3.21 (0.62)	0.443	

Note: [a] Scores from 'not received' to 'would like more training', score 1 to 3; [b] Scores from 'Not at all' to 'Extremely often', score 1-4; * $P < 0.05$; [c] At least two items answered.

Table S2. Correlations matrix between variables with significant correlation with psychological distress.

	1	2	3	4	5	6	7	8	9	10	11
1. Training: COVID-19 infection control	1										
2. Training: Emergency Procedures	**.187***	1									
3. JRTI	0.038	**-.195***	1								
4. Perceived risks of COVID-19	-0.147	-0.138	**0.509****	1							
5. Social support: immediate supervisor/boss	0.123	0.158	**-0.320****	**-0.247****	1						
6. Workplace hazards: hazardous in general	0.088	0.103	**-0.251****	**-.330****	0.143	1					
7. Workplace hazards: hazardous since the pandemic	0.031	0.214*	-0.170	**-0.231***	-0.005	**0.455****	1				
8. Compliance: protective practices – COVID-19	**0.218****	0.072	0.032	-0.002	0.048	0.123	0.132	1			
9. Compliance: protective practices - Non-COVID-19	**0.194***	0.114	0.003	-0.065	**0.221***	0.144	0.141	**0.631****	1		
10: Workplace relationship	**0.204***	**0.252****	-0.175	-0.148	**0.365****	**0.188***	**0.197***	**0.599****	**0.519****	1	
11. Perception of barriers to infection control practices	-0.129	-0.181	0.138	**0.228***	**-0.319****	**-0.224***	**-0.266****	-0.170	**-0.267****	**-0.370****	1

Note: * Pearson's correlation coefficient is significant at the 0.05 level (2-tailed); ** Pearson's correlation coefficient is significant at the 0.01 level (2-tailed).

Table S3. Results for mediation analysis with psychological distress as a dependent variable and perceived risks of COVID-19 as a mediator.

	Consequent					
	M (Perceived risks)			Y (PD)		
**Antecedent**	**Coefficient β**	**SE**	**P**	**Coefficient β**	**SE**	**P**
**X (JRTI)**	0.56	0.11	0.00**	0.49	0.32	0.13
**M (Perceived risks)**				0.51	0.24	0.03*
**Direct effect (X --> Y)**	Effect: 0.49; SE: 0.32; P = 0.13					
**Indirect effect (X --> M --> Y)**	Effect: 0.29; BootSE: 0.14; BootCI (0.04, 0.61)[a]					

Note: The above results are expressed in a log-odds metric; X: predictor;
Y: dependent variable; M: mediator; SE: standard error; BootSE: bootstrap SE;
BootCI: bootstrap confidence interval; Covariates: immediate supervisor/boss support and concern about the hazardous workplace in general; * P < 0.05; ** P < 0.01; [a]Statistically significant.

Table S4. Results from a regression analysis examining the moderation effect of 'workplace relationship' on 'compliance with protective practices for COVID-19 and Non-COVID-19 patients' to 'psychological distress.'

Variables	Coefficient β	SE	P
**Psychological Distress (Y)**			
**Compliance COVID-19 (X1)**	0.04	0.41	0.928
**Workplace Relationship (W)**	-0.98	0.41	0.017*
**Compliance COVID-19 x Workplace Relationship (X1*W)**	-0.79	0.29	0.007**
**Likelihood ratio test of the highest order unconditional interactions X1*W:**	Chi-square: 7.81; df = 1; P = 0.005**		
**Psychological Distress (Y)**			
**Compliance Non-COVID-19 (X2)**	0.25	0.34	0.46
**Workplace Relationship (W)**	-0.93	0.42	0.025*
**Compliance Non-COVID-19 x Workplace Relationship (X2*W)**	-0.99	0.31	0.001**
**Likelihood ratio test of the highest order unconditional interactions X2*W:**	Chi-square: 11.10; df = 1; P = 0.001**		

Note: The above results are expressed in a log-odds metric; X1 & X2: predictors; Y: dependent variable;
W: moderator; SE: standard error; Covariates: Compliance with protective practices for COVID-19 or Non-COVID-19 patients, immediate supervisor/boss support and perceived barriers to infection control; * P < 0.05; ** P < 0.01.

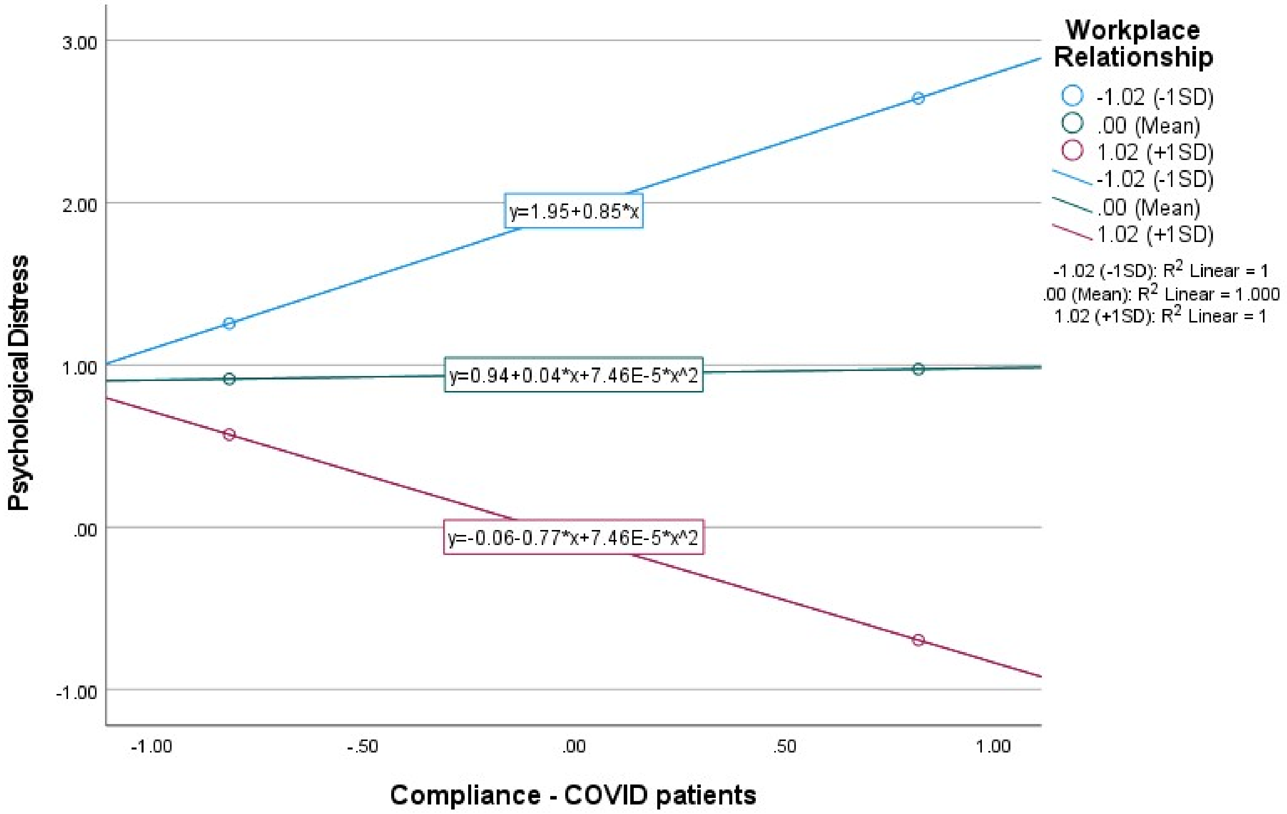

Note: SD: Standard Deviation; Compliance–COVID-19 Patients: compliance with protective practices for COVID-19 patient care.

Figure S1. Visualization of the conditional effect of the predictor (Compliance–COVID-19 patients) at values of the moderator (Workplace Relationship) when the Workplace Relationship mean score is at 1 SD below the mean, mean, and 1 SD above the mean value.

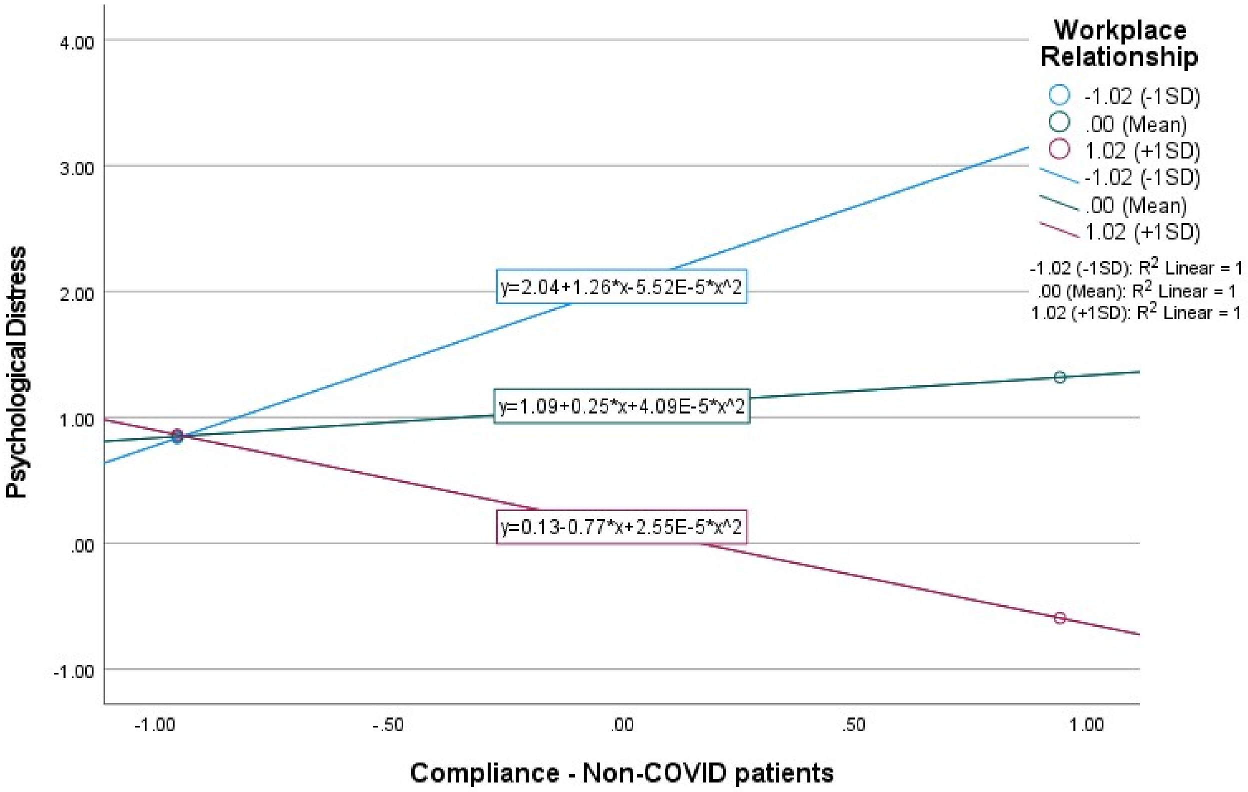

Note: SD: Standard Deviation; Compliance–Non-COVID-19 Patients: compliance with protective practices for Non-COVID-19 patient care.

Figure S2. Visualization of the conditional effect of the predictor (Compliance–Non-COVID-19 patients) at values of the moderator (Workplace Relationship) when the Workplace Relationship mean score is at 1 SD below the mean, mean, and 1 SD above the mean value.

STROBE Statement—Checklist of items that should be included in reports of ***cross-sectional studies***

	Item No	Recommendation	Page No
**Title and abstract**	1	(*a*) Indicate the study's design with a commonly used term in the title or the abstract	1
		(*b*) Provide in the abstract an informative and balanced summary of what was done and what was found	1
**Introduction**			
Background/rationale	2	Explain the scientific background and rationale for the investigation being reported	1-2
Objectives	3	State specific objectives, including any prespecified hypotheses	2
**Methods**			

Study design	4	Present key elements of study design early in the paper	3
Setting	5	Describe the setting, locations, and relevant dates, including periods of recruitment, exposure, follow-up, and data collection	3
Participants	6	(*a*) Give the eligibility criteria, and the sources and methods of selection of participants	3
Variables	7	Clearly define all outcomes, exposures, predictors, potential confounders, and effect modifiers. Give diagnostic criteria, if applicable	3-4
Data sources/ measurement	8*	For each variable of interest, give sources of data and details of methods of assessment (measurement). Describe comparability of assessment methods if there is more than one group	4-5
Bias	9	Describe any efforts to address potential sources of bias	19
Study size	10	Explain how the study size was arrived at	6
Quantitative variables	11	Explain how quantitative variables were handled in the analyses. If applicable, describe which groupings were chosen and why	4-5
Statistical methods	12	(*a*) Describe all statistical methods, including those used to control for confounding	5
		(*b*) Describe any methods used to examine subgroups and interactions	5
		(*c*) Explain how missing data were addressed	5
		(*d*) If applicable, describe analytical methods taking account of sampling strategy	5
		(*e*) Describe any sensitivity analyses	5
**Results**			
Participants	13*	(a) Report numbers of individuals at each stage of study–e.g., numbers potentially eligible, examined for eligibility, confirmed eligible, included in the study, completing follow-up, and analysed	6
		(b) Give reasons for non-participation at each stage	6
		(c) Consider use of a flow diagram	n/a
Descriptive data	14*	(a) Give characteristics of study participants (e.g., demographic, clinical, social) and information on exposures and potential confounders	6-7
		(b) Indicate number of participants with missing data for each variable of interest	6-7
Outcome data	15*	Report numbers of outcome events or summary measures	7-15
Main results	16	(*a*) Give unadjusted estimates and, if applicable, confounder-adjusted estimates and their precision (e.g., 95% confidence interval). Make clear which confounders were adjusted for and why they were included	7-15
		(*b*) Report category boundaries when continuous variables were categorized	7-15
		(*c*) If relevant, consider translating estimates of relative risk into absolute risk for a meaningful time period	n/a

Other analyses	17	Report other analyses done–e.g., analyses of subgroups and interactions, and sensitivity analyses	13-15
**Discussion**			
Key results	18	Summarise key results with reference to study objectives	15-18
Limitations	19	Discuss limitations of the study, taking into account sources of potential bias or imprecision. Discuss both direction and magnitude of any potential bias	19
Interpretation	20	Give a cautious overall interpretation of results considering objectives, limitations, multiplicity of analyses, results from similar studies, and other relevant evidence	15-18
Generalisability	21	Discuss the generalisability (external validity) of the study results	19
**Other information**			
Funding	22	Give the source of funding and the role of the funders for the present study and, if applicable, for the original study on which the present article is based	20

*Give information separately for exposed and unexposed groups.

Note: An Explanation and Elaboration article discusses each checklist item and gives methodological background and published examples of transparent reporting. The STROBE checklist is best used in conjunction with this article (freely available on the Web sites of PLoS Medicine at http://www.plosmedicine.org/, Annals of Internal Medicine at http://www.annals.org/, and Epidemiology at http://www.epidem.com/). Information on the STROBE Initiative is available at www.strobe-statement.org.
